# Sitosterolemia misdiagnosed as homozygous familial hypercholesterolemia: A diagnostic challenge

**DOI:** 10.1016/j.ajpc.2026.101651

**Published:** 2026-04-22

**Authors:** Anthony Matta, Vanessa Nader, Dorota Ferrières, Jean Ferrières

**Affiliations:** a Department of cardiology, Centre Hospitalier Intercommunal des Vallées de l’Ariège, Saint Jean de verges, France; b Department of cardiology, Toulouse Rangueil University Hospital and UMR INSERM 1295, TSA 50032 Toulouse, France; c Notre Dame des Secours University Hospital Center, Street 93, Byblos, Lebanon, P.O.Box 446, School of Medicine and Medical Sciences, Holy Spirit University of Kaslik, Jounieh, Lebanon; d Inserm I2MC-UMR1297, Toulouse, France

**Keywords:** Sitosterolemia, Familial hypercholesterolemia, Sterols

## Abstract

Sitosterolemia is a rare genetic disease caused by loss of function homozygous or compound heterozygous mutations in either ABCG5 or ABCG8 genes encoding sterols transporters. The net effect leads to an increase in sterols intestinal absorption and a reduction in sterols bile excretion causing sterols accumulation in plasma and tissues. The spectrum of clinical manifestations is variable and sitosterolemia is often misdiagnosed as familial hypercholesterolemia, particularly in patients with tendon or tuberous xanthomas. However, the triad of hematologic abnormalities, good response to ezetimibe therapy and poor response to statins should raise the suspiscion for sitosterolemia. The diagnosis is based on high plasma sterols (sitosterol and campesterol) -the hallmark laboratory feature of sitosterolemia- and confirmed by genetic analysis. The combination of dietary approach and ezetimibe therapy represents the mainstay treatment of sitosterolemia patients. Lipoprotein apheresis could be used as adjunctive therapy. This case illustrates a stepwise approach for the diagnosis of sitosterolemia and summarizes the pathophysiology, clinical presentations and management of sitosterolemia. It raises sitosterolemia awareness among physicians for an early diagnosis and appropriate therapeutic approach.

## 1 Introduction

Sitosterolemia, also known as phytosterolemia or xenosterolemia, is a rare inborn autosomal recessive lipid metabolic disorder firstly described by Connor and Bhattacharyya in 1974 [1]. This genetic condition is caused by homozygous or compound heterozygous biallelic loss of function mutations in either ABCG5 or ABCG8 encoding sterol efflux transporters, thereby accumulating phytosterols in blood and tissues because of an increased intestinal absorption and decreased biliary excretion of plant sterols [2]. Sitosterolemia encompasses diverse phenotypes and a wide spectrum of clinical manifestations, which can make diagnosis challenging. Hematologic manifestations including hemolysis, macrothrombocytopenia, splenomegaly, and abnormal bleeding, arthralgia, arthritis and liver failure were reported in sitosterolemia patients. They are frequently misdiagnosed as familial hypercholesterolemia, particularly in patients presenting with tendinous or tuberous xanthomas. Clinical presentation depends on dietary sterol intake and ranges from an asymptomatic state to premature coronary atherosclerosis and cardiac death. A marked improvement of hypercholesterolemia following dietary modifications or ezetimibe/bile acid sequestrants therapy, together with a poor response to statins, represent a key diagnostic feature of sitosterolemia [3]. Diagnostic challenges and the potential for misdiagnosis as familial hypercholesterolemia make it difficult to determine the true prevalence of this metabolic disorder, with approximately 200 cases reported so far [4]. Raising awareness of its characteristics among healthcare professionals may facilitate accurate and early diagnosis, leading to appropriate treatment and improved prognosis. This review presents and summarizes its clinical manifestations, pathophysiology, diagnostic procedure, and treatment strategies.

## 2 Clinical case

A 61-year-old woman presented to the department of preventive cardiology at Toulouse university hospital for cardiac follow up after moving to this region. She was diagnosed in childhood with homozygous familial hypercholesterolemia with an initial total cholesterol level of 680 mg/dl and tendinous xanthomas. She was initially treated with clofibrate, which reduced her total cholesterol level to 300 mg/dl. After clofibrate therapy, she received Cholestyramine for several years. Statin therapy was discontinued because of an unexplained decrease in the platelets count during treatment. Then, she started ezetrol 10 mg, which effectively lowered the total cholesterol level to 269 mg/dl and low-density cholesterol level to 188 mg/dl. She was referred to LDL-c apheresis until 2018, when therapy was switched to biweekly alirocumab 150 mg injections. Her family history is notable for two brothers who died at a young age. The first died at 15 years of age during coronary angiography and had a markedly elevated total cholesterol level of 400 mg/dl. The second died at age of 20 from sudden cardiac death. Her daughter had a high total cholesterol level. Her medical history included surgical replacement of the aortic valve with a mechanical prosthesis at 50 years of age for severe aortic stenosis. The absence of high cholesterol level in her parents and the good response to ezetrol raised suspicion for a differential diagnosis rather than homozygous familial hypercholesterolemia. The investigations were completed by genetic analysis of genes encoding LDL-C receptors, Apolipoprotein B, Apolipoprotein E and PCSK9 and did not reveal any pathogenic variants. The diagnostic workup was advanced by quantifying plasma sterols (sitosterol and campesterol) followed by sequencing the ABCG5 gene. An elevated level of sterol (465 μmol/L; upper control limit 8 μmol/L) and campesterol (242 μmol/L; upper control limit 10.6 μmol/L) were found, and two pathogenic variants in exons 3 (c.338T>G, p. Val113Gly) and 6 (c.656C>G, p. Pro219Arg) of ABCG5 were identified. The diagnosis of sitosterolemia was confirmed, indicating that the patient had been misdiagnosed with homozygous familial hypercholesterolemia for >50 years. The hematologic abnormalities initially observed and attributed to statin therapy were also likely linked to sitosterolemia. Ezetrol therapy, initiated decades earlier, together with the healthy lifestyle and Mediterranean diet followed by the patient, may explain the absence of severe cardiovascular complications. Patient was referred to dietitian to control dietary sterol intake, the key role in clinical manifestations, complications development and case management. Her daughter was also addressed for genetic testing.

## 3 Discussion

**Fig. 1:**
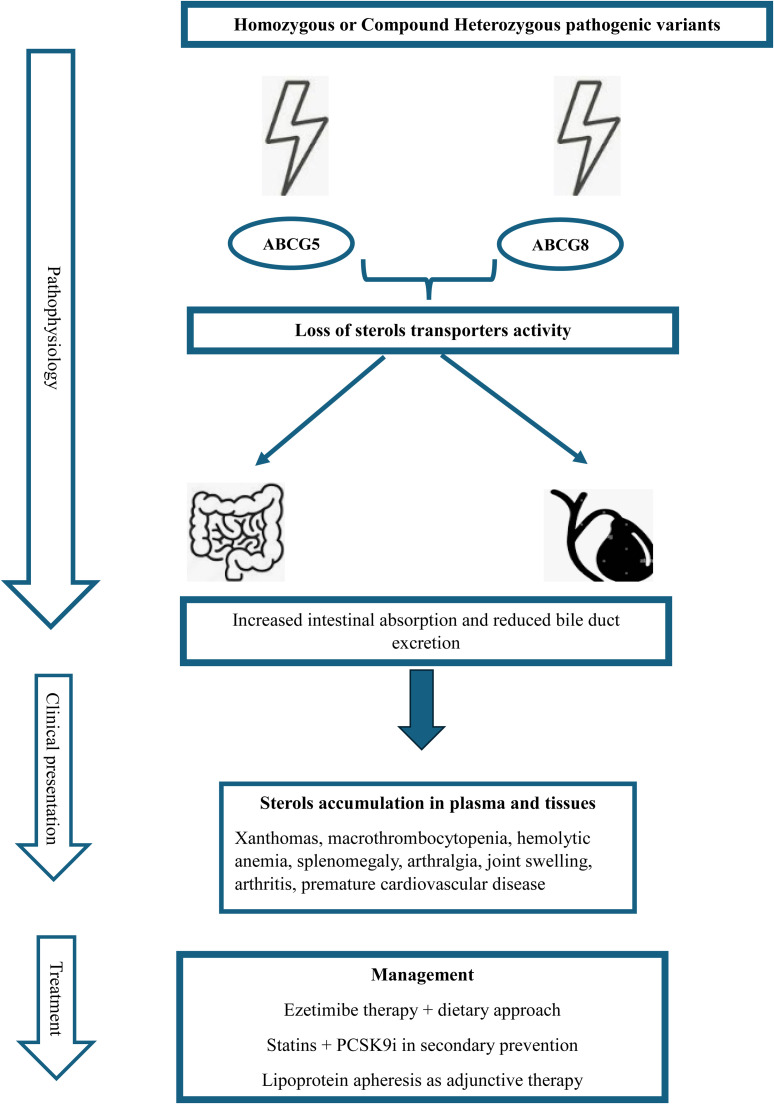
Pathophysiology of sitosterolemia.

Sitosterolemia is a genetic disease caused by a loss of function mutation in the ABCG5 or ABCG8 gene encoding sterols transporters (sterolin-1 and sterolin-2) responsible for the transport of sterols to the bile ducts and intestinal lumen for elimination [2]. These mutations are mainly homozygous but may also occur as compound heterozygous variants. Table 1 showed the list of mainly reported mutations in sitosterolemia. In this case, we found two pathogenic variants in exons 3 and 6 of ABCG5 gene [[5], [6]]. Loss of ABCG5/ABCG8 transporter activity increases the intestinal absorption of dietary plant sterols and reduces their biliary excretion leading to plasma and tissues accumulation of phytosterols such as sitosterol and campesterol. The increased intestinal absorption of sterols increases the hepatic cholesterol pool and the increased sterol flux through the liver indirectly increases LDL-c formation. Thus, LDL-c elevation is highly variable among sitosterolemia patients. Also, LDL particles in sitosterolemia carry abnormally high phytosterol content which may enhance their atherogenic potential. However, the causal link between a high plasma phytosterols level and atherosclerosis is unclear and not well understood [7]. Plant sterols incorporate into erythrocyte and platelet membranes, altering membrane fluidity and structure. This may cause hemolytic anemia or macrothrombocytopenia or abnormal red cell morphology (stomatocytosis) [8] (Fig. 1).

**Table 1:** Types of genetic mutations reported in ABCG5 and ABCG8 genes.

Type of mutation	ABCG5 gene	ABCG8 gene
**Missense mutations**	p.Arg145Cys(c.433C>T) p.Arg389His(c.1166G˃A) p.Arg419His(c.1256G˃A) p.His510Asn(c.1568C˃A) p.Ser44Pro(c.130T˃C) p.Val113PGly(c.338T˃G) p.Pro219Arg (c.656C˃G)	p.Asp19His(c.55G˃C) p.Arg121Gln(c.362G˃A) p.Ile419Asn(c.1256T˃A) p.Gly432Val(c.1295G˃T) p.Leu195Gln(c.584T˃A)
**Nonsense mutations**	p.Glu22Ter(c.64C˃T) p.Gln251Ter(c.751C˃T) P.Arg446Ter	p.Glu217Ter(c.647dup) p.Tyr303Ter(c.909T˃G) p.Trp361Ter p.Arg412Ter
**Splice-site mutation**	c.904+1G˃A	IVS1–2A˃G
**Frameshift mutations**	p.Pro558GlnfsTer14 (c.1673_1677del) c.336–337insA

The clinical presentation of sitosterolemia is variable and heterogeneous. The onset and severity of symptoms often correlate with dietary sterol intake. Reported manifestations include integumentary, cardiovascular, musculoskeletal and hematological systems. One of the most common features is the presence of tendon xanthomas in childhood, usually associated with elevated cholesterol level [9]. These findings may lead to misdiagnosis of familial hypercholesterolemia and contribute to the development of premature atherosclerotic cardiovascular disease [8]. An observational study reported the prevalence of 93% of tendon and skin xanthomas in pediatric population [10]. A 1.8-fold increase in the risk of major coronary events were reported in sitosterolemia patients with plasma sitosterol concentrations (>5.25µmol/l) [11]. Myocardial infarction and sudden cardiac death have been reported during childhood or early adulthood (5–18 years), especially in untreated patients [9]. This case clearly demonstrates that high cholesterol level and tendon xanthomas contributed to the initial misdiagnosis, whereas the lack of early cardiovascular events may be attributable to ezetimibe therapy together with a well-controlled diet. Sterol accumulation in synovial and periarticular tissues causes tendon thickening, arthralgia, joint swelling and arthritis [8]. Hematologic abnormalities ensuing from sterols deposition in red blood cells, platelets and bone marrow are very common in sitosterolemia (up to 60%). Macrothrombocytopenia is a hallmark hematologic characteristic, and other abnormalities include hemolytic anemia, splenomegaly and stomatocytosis [9]. It is noteworthy that hematologic features improve with ezetimibe therapy as shown in this case.

The diagnosis of sitosterolemia is based on four major criteria: clinical suspicion, laboratory testing, genetic analysis and differential diagnosis. Xanthomas are the main clinical features of sitosterolemia, familial hypercholesterolemia and cerebrotendinous xanthomatosis. However, the cholesterol level differs among these conditions: it is highly variable in sitosterolemia, consistently elevated in familial hypercholesterolemia and typically normal or mildly increased in cerebrotendinous xanthomatosis [12]. The hematologic abnormalities such as red blood cells morphology or thrombopenia are more characteristic of sitosterolemia, whereas neurological symptoms are more typical for cerebrotendinous xanthomatosis. The triad of xanthomas, hematologic abnormalities, and good response to ezetimibe therapy should raise the clinical suspicion of sitosterolemia. The laboratory hallmark for sitosterolemia is high plasma sterols level (campesterol and sitosterol) [13], whereas a high cholestanol level is typical for cerebrotendinous xanthomatosis [13]. To confirm the diagnosis, genetic analysis reveals causative mutations in genes encoding ABCG5 or ABCG8 for sitosterolemia, CYP27A1 for cerebrotendinous xanthomatosis and LDL-receptors, Apolipoprotein B, Apolipoprotein E or PCSK9 for familial hypercholesterolemia (Table 2).

**Table 2:** Differential diagnosis in patients with xanthomas.

Feature	Sitosterolemia	Familial hypercholesterolemia	Cerebrotendinous xanthomatosis
**LDL-c level**	Variable (mildly-to- moderately increased)	Markedly increased	Usually normal or mildly increased
**Commonly associated clinical signs**	Hematological abnormalities		Neurological abnormalities
**Hallmark biological parameter**	Elevated sitosterol and campesterol plasma level	High LDL-c level	Elevated cholestanol plasma level
**Causative mutations genes**	ABCG5 or ABCG8	LDL-C receptors or Apo-B or Apo-E or PCSK9	CYP27A1
**Treatment**	Sterols restriction + ezetimibe therapy	Statins + ezetimibe + PCK9i + bempedoic acid	Chenodeoxycholic acid

**Fig. 2:**
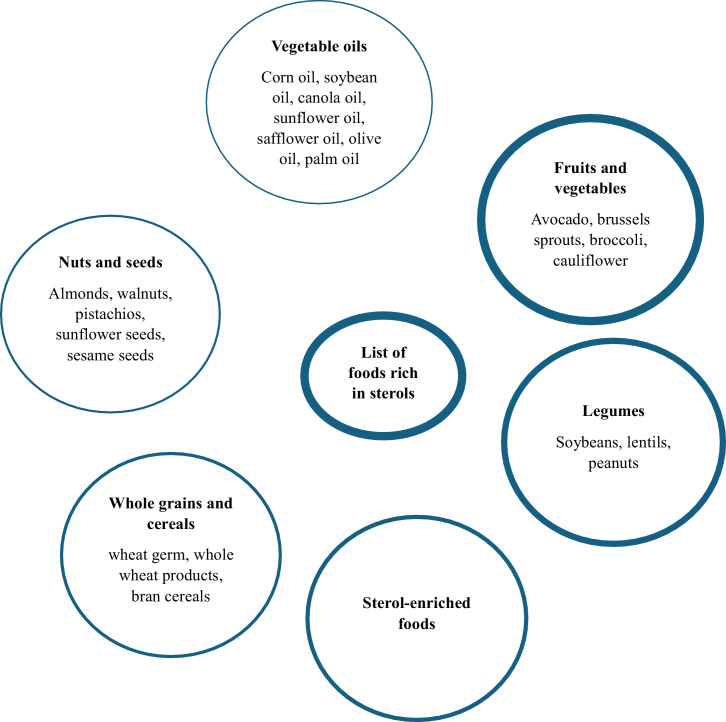
List of food rich in sterols.

The combination of dietary approach and ezetimibe therapy represents the mainstay treatment of sitosterolemia patients [3]. The goal of dietary approach is to reduce plant sterols intake as well as cholesterol. Fig. 2 showed the list of plant rich in sterols [[14], [15]]. Ezetimibe, an inhibitor of cholesterol intestinal absorption, effectively reduces plasma plant sterols in patients with sitosterolemia. A 21% decrease in sitosterol concentration and 24% reduction in campesterol level were observed after 8 weeks treatment with ezetimibe [16]. The net effect leads to a progressive reduction in xanthoma thickness and improvement in platelets count and hematocrit level [17]. Higher rate of reduction in plasma concentrations of sitosterol (43.9%) and campesterol (50.8%) was observed up to 2 years with ezetimibe therapy [17]. In sitosterolemia, statins use is not effective and does not decline plasma sterols level because HMG-CoA reductase activity is already maximally inhibited [18]. However, statins and PCSK9 inhibitors could be used in the setting of secondary prevention. Lastly, lipoprotein apheresis can be used as adjunctive therapy when first-line treatments are ineffective or in severe and refractory cases [19]. Genetic counseling plays an important role in evaluating relatives at risk because early diagnosis either through measurement of plasma sterols concentrations or through molecular genetic testing (if the family-specific pathogenic variants are known) allows early introduction of treatment and surveillance to optimize outcome and prevent serious complications like premature atherosclerotic cardiovascular disease [20].

## 4 Conclusion

Sitosterolemia is a rare genetic metabolic lipid disorder characterized by a markedly elevated plasma sterols and well responding to both dietary restrictions and ezetimibe therapy. Early diagnosis is crucial for beginning the appropriate treatment to prevent complications onset like premature atherosclerotic cardiovascular disease. However, making the diagnosis is particularly challenging given the wide and often non-specific clinical spectrum. Many patients with sitosterolemia may be misdiagnosed for a long time as having familial hypercholesterolemia, leading to a missed or delayed accurate diagnosis. An example is the reported case where the patient was misdiagnosed and treated for familial hypercholesterolemia for over five decades. The presence of hematologic abnormalities in patients with cutaneous or tuberous xanthoma, along with a lack of response to statins therapy, should be considered key features that raise suspicion of sitosterolemia. This case increases the awareness of sitosterolemia among physicians and provides an overview of differential diagnostic clues and therapeutic approaches, with an emphasis on understanding the underlying pathophysiology.

## Author agreement statement

We the undersigned declare that this manuscript is original, has not been published before and is currently not being considered for publication elsewhere. We confirm that the manuscript has been read and approved by all named authors and that there are no other persons who satisfied the criteria for authorship but are not listed. We further confirm that the order of authors listed in the manuscript has been approved by all of us. We understand that the Corresponding Author is the sole contact for the Editorial process. He is responsible for communicating with the other authors about progress, submissions of revisions and final approval of proofs Signed by all authors as follows:

-Anthony Matta

- Dorota Ferrières

-Vanessa Nader

-Jean Ferrières

## CRediT authorship contribution statement

**Anthony Matta:** Writing – original draft, Validation, Data curation, Conceptualization. **Vanessa Nader:** Writing – original draft, Validation, Conceptualization. **Dorota Ferrières:** Writing – original draft, Validation, Conceptualization. **Jean Ferrières:** Writing – original draft, Validation, Supervision, Conceptualization.

## Declaration of competing interest

The authors declare that they have no known competing financial interests or personal relationships that could have appeared to influence the work reported in this paper.
